# Compact high-resolution FBG strain interrogator based on laser-written 3D scattering structure in flat optical fiber

**DOI:** 10.1038/s41598-023-35708-1

**Published:** 2023-05-31

**Authors:** Przemyslaw Falak, Timothy Lee, Shahrzad Zahertar, Bo Shi, Bruno Moog, Gilberto Brambilla, Christopher Holmes, Martynas Beresna

**Affiliations:** Optoelectronics Research Centre, University of Southampton, Southampton, SO17 1BJ UK

**Keywords:** Laser material processing, Optical metrology, Optical sensors

## Abstract

We demonstrate a fiber Bragg grating (FBG) strain interrogator based on a scattering medium to generate stable and deterministic speckle patterns, calibrated with applied strain, which are highly dependent on the FBG back-reflection spectral components. The strong wavelength-dependency of speckle patterns was previously used for high resolution wavemeters where scattering effectively folds the optical path, but instability makes practical realization of such devices difficult. Here, a new approach is demonstrated by utilizing femtosecond laser-written scatterers inside flat optical fiber, to enhance mechanical stability. By inscribing 15 planes of pseudo-randomized nanovoids (714 $\times$ 500 voids per plane) as a 3D array in a 1 $\times$ 0.7 $\times$ 0.16 mm volume, the intrinsic stability and compactness of the device was improved. Operating as a wavemeter, it remained stable for at least 60 h with 45 pm resolution over the wavelength range of 1040–1056 nm. As a reflection mode FBG interrogator, after calibrating speckle patterns by applying tensile strain to the FBG, the device is capable of detecting microstrain changes in the range of 0–200 $\mu \epsilon$ with a standard error of 4 $\mu \epsilon$, limited by the translation stage step size. All these characteristics make it an interesting technology for filling the niche of low-cost, high-resolution wavemeters and interrogators which offer the best available trade-off between resolution, compactness, price and stability.

## Introduction

**Figure 1:**
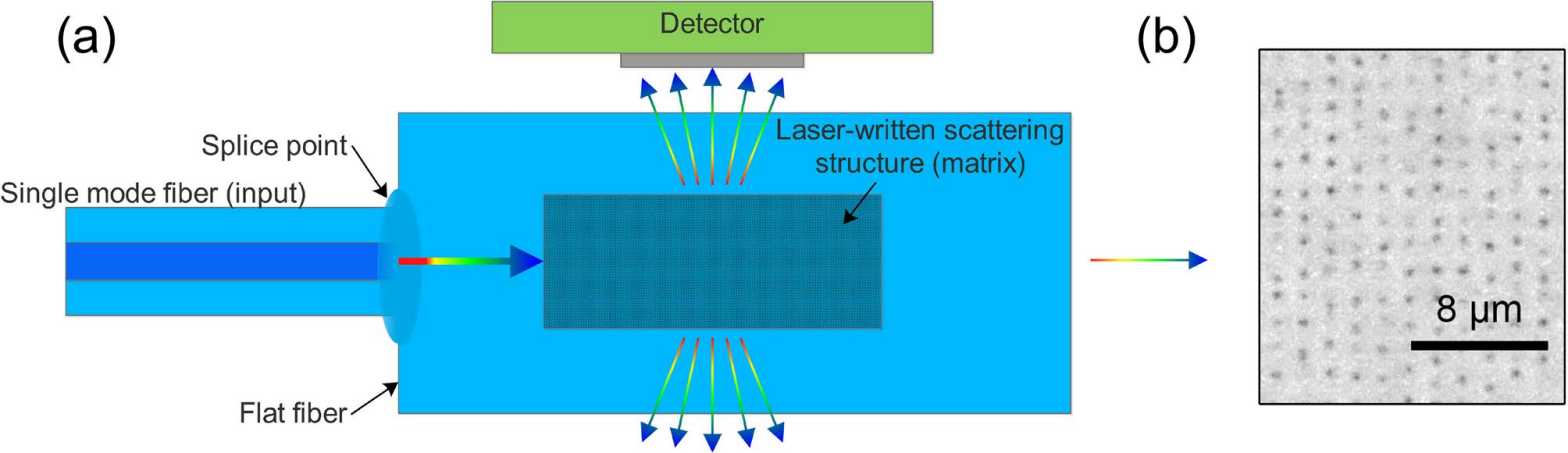
(**a**) Schematic of flat fiber-inscribed scattering/multimodal interference structure. Light enters via single mode fiber into flat fiber, then diffracts and couples into various flat fiber modes. Once it reaches the scattering matrix (dashed structure), the light is scattered and speckles are imaged at the detector. Ballistic light exits from the right side and is not traced by the detector. Elements not to scale. (**b**) Microscope image of laser-written nanovoids array within flat fiber scattering structure.

There has been extensive research and development on fiber Bragg gratings (FBG) as sensors in many industries, including civil engineering, aeronautics, and telecommunications, due to their mature fabrication, high sensitivity, ease of multiplexing and immunity to electromagnetic interference^1–3^. Here, we demonstrate an FBG interrogator for tensile strain measurements, based on analysing the speckle patterns generated by the FBG backreflected light. This spectral-to-spatial mapping paradigm was previously exploited by reconstructive wavemeters using various scattering or interference media^4–8^ to generate deterministic and spectrally unique speckle patterns. We developed a 3D array of scattering nanovoids, inscribed inside a flat fiber which acts as a highly stable scattering medium suitable for a fine resolution wavemeter and interrogator as shown in Fig. 1. The speckle patterns, which are the planar projections of mutual interference of light from different scattering points, are unique for any given wavelength with a one-to-one mapping. Therefore, to operate as a wavemeter, the calibration set of the speckles for given wavelengths can be created by tuning a laser source wavelength, and then an unknown wavelength signal in the calibration range can be reconstructed by solving linear algebra correlation equations^5,9–12^.

Confining the optical path in the compact scattering medium reduces cost, complexity and device footprint due to an easier and lower-cost fabrication of the scattering system where only a detector and scattering medium are needed. This is in contrast to conventional wavemeters/spectrometers, which require a dispersive medium to spatially separate wavelength components onto a detector; such systems utilize bulk prisms or gratings with additional components (like monochromators) and linear detectors, resulting in more complexity, higher cost in fabrication and also larger device size since a longer path length is required for finer resolution. Although there is a clear trend towards miniaturization of dispersive medium-based devices^13–16^, there remain clear trade-offs between resolution, device size and cost.

Since the reconstructive device captures wavelength-dependent speckle patterns, its use as an FBG interrogator is also demonstrated. Conventional FBG interrogation involves launching light from a broadband or tunable source onto the FBG and then detecting the backreflected signal (Bragg resonance wavelength $\lambda _B$). Since $\lambda _B$ is dependent on the grating periodicity and effective refractive index, applying strain will generally induce a redshift in $\lambda _B$^17,18^. For reconstructive systems, the shift in $\lambda _B$ causes the speckle pattern motif change. The same principle would apply to multiple FBGs on the same fiber, since the speckle changes can be decomposed into single-FBG constituents. Moreover, to reconstruct the FBG strain, only changes of the corresponding speckles need to be measured, since these would be calibrated directly to strain, and knowledge of the spectral shift value is not required.

Conventional FBG interrogators have the same challenges to overcome as dispersive spectrometers, since the interrogation process requires the spectral resolution to be extremely high, down to picometers. In addition, such devices are increasingly utilized in extreme environment conditions (such as aviation and construction), requiring not only compactness and high resolution, but also stability and durability to the external environment. Therefore, there is an opportunity to engineer a reconstructive device meeting these demands in the form of an innovative FBG strain interrogator based on spectral-to-spatial mapping.

Scattering-based reconstructive devices require the speckles to remain unchanged for the calibrated input wavelengths or spectra in order to successfully reconstruct an unknown spectrum. However, the fluctuating environment may change the speckles generated and hence affect the whole measurement process. That is why the engineering of intrinsically stable devices and instability compensation has become a focal point in the development of miniaturized spectrometers.

Previous reports of scattering or interference media for generating speckle patterns used alumina powder^19^, integrating spheres^20,21^, multi-mode fiber (MMF)^5,22^, 2D^9^ or 3D laser-written scattering chips^23^. Whilst most of the reported devices offered increased compactness and fine resolution (ranging from nm to pm), their stability was limited from only a few minutes up to a few hours depending on the applied medium. This work considers a flat silica optical fiber as the substrate for the scattering medium to solve this issue^24,25^. In contrast to circular fibers of a similar thickness, the rectangular cross-sectional geometry provides a stiffer configuration less prone to accidental bending, twisting or stretching, contributing towards intrinsic stability. It is also fully compatible with regular optical fibers, in that it can be fusion spliced to it. Furthermore, due to the lack of curvature on the top face of the fiber, light is not lensed, and thus the speckles are less distorted. Femtosecond laser writing was chosen due to its ability to create nanoscale voids in the material acting as Mie scattering centers. In addition, the process can be fully engineered, is repeatable and allows writing the structures not only on the surface, but embedded up to hundreds of microns inside the substrate, which in turn increases stability and durability of the device.

In previous work, the best compromise between device stability and spectral resolution was established by engineering a $20 \times 20$ $\upmu$m scattering chip which provided a resolution as small as 0.75 nm^26^. The only major disadvantage was the inevitable loss of light out of the main scattering plane. By creating laser-written, randomly-distributed arrays of nanovoids in spatially separated planes, a 3D randomized array structure, where the optical path of light is folded multiple times, provided an enhanced speckle wavelength dispersion and reduced out-of-plane losses. This 3D chip-based wavemeter/spectrometer^23,27^, which utilized a $1 \times 1 \times 0.02$ mm scattering pattern inscribed within $10 \times 10 \times 1$ mm fused silica plate, was mounted inside a plastic monocoque enclosure between an input beam collimator on one side and the camera sensor on the other. Whilst the 3D chip demonstrated improved intrinsic stability with environment and time, the plastic enclosure was contracting/expanding in line with the temperature fluctuations in the lab. Such changes caused displacement between the chip and the detector. The resulted speckles motifs remained the same, but were subjected to a translational movement. Such instability was compensated by pixel binning the speckle images, giving a resolution up to 50 pm with 170 hours stability. The key disadvantage was low sensitivity due to light scattering over a wide area, of which only a portion is captured by the sensor.

Whilst the 3D chip was more stable, the MMF-based system still offered finer spectral resolution. To combine the advantages of both approaches, in this work the scattering array (matrix) is inscribed in a flat fiber to which an input single-mode fiber pigtail is fused. Speckle patterns are generated by a combination of multimodal interference and Mie scattering. This solves the problem with instability, but also further reduces device size and cost, as the fiberized input does not require collimators. Furthermore, the camera detector is now placed perpendicularly to the ballistic light propagation direction, thus it avoids speckle overexposure caused by the unscattered beam. Speckles of neighbouring wavelengths are also more distinguishable, since the ballistic background is eliminated and only high-angled scattering pathways are allowed to reach the detector (Fig. 1).

## Results

### Wavemeter

**Figure 2:**
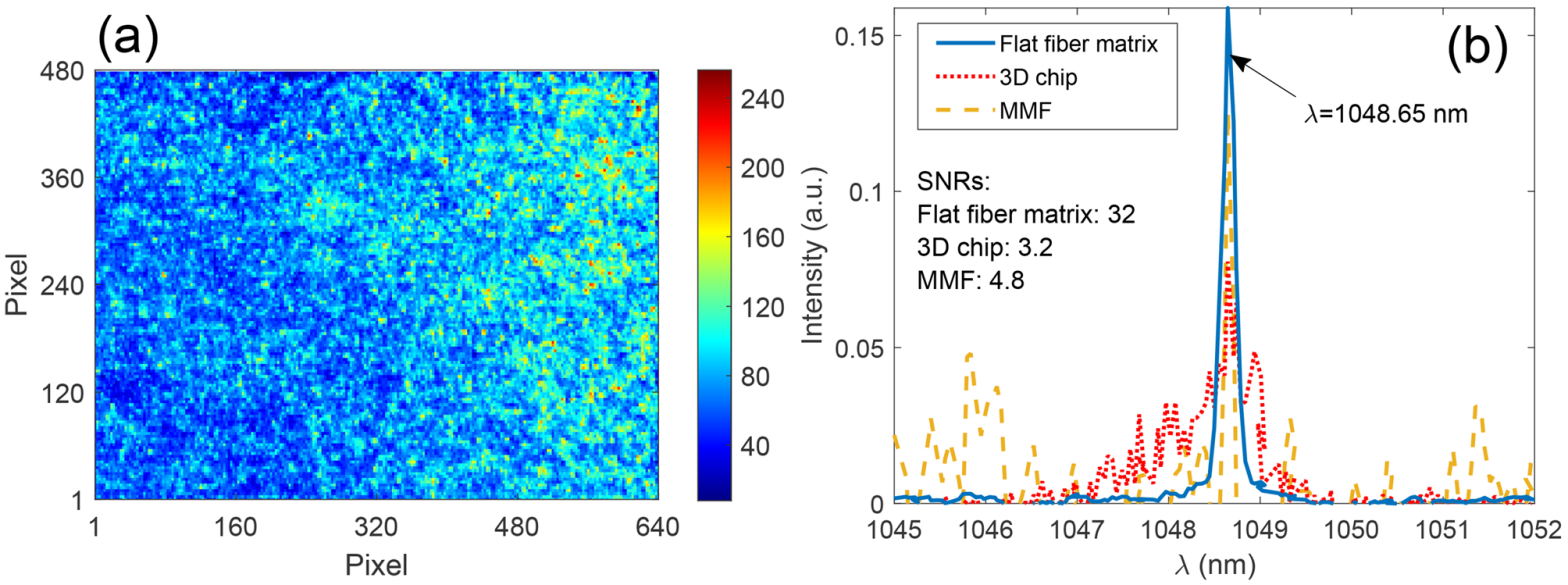
(**a**) $640 \times 480$ pixels speckle pattern (colorbar corresponds to 8-bit pixel values 0–255). (**b**) Signal-to-noise ratio (SNR) comparison between flat fiber-inscribed scattering matrix, 3D chip and MMF-based wavemeter for fixed single wavelength input $\lambda =1048.65$ nm.

The key operating principle for reconstructive scattering wavemeters is finding ‘weights’—correlation parameters (also known as intensities) which indicate how much of each speckle from the calibration set (and thus of each wavelength) forms the measured spectrum speckle. Depending on the scattering medium system, the reconstructed single-wavelength spectrum can be characterized by different peak shape, width and base noise level, which will impact the performance metrics: signal-to-noise ratio (SNR) and spectral resolution. The exemplar speckle and the results of single-wavelength ($\lambda =1048.65$ nm) spectral reconstruction for 3 different scattering structures are compared in Fig. 2: the flat fiber-based scattering matrix, a 50 cm section of MMF and a 3D scattering chip.

Indeed, each of the cross-compared devices produces differently-shaped spectra for the same input wavelength. The MMF-based system (Fig. 2b, dashed line) exhibits a sharp central peak with an intensity of 0.13 and full-width half-maximum (FWHM) of $\sim$ 0.10 nm; however, due to the high background noise (on average, 0.027) caused by the instability of the fiber during calibration, its SNR was only 4.8. The 3D scattering chip-based device spectrum (Fig. 2b, dotted line) shows a lower noise level (averaged 0.025), but the peak is broader (FWHM $\sim$ 0.9 nm) and its intensity lower ($\sim$ 0.08), reducing the SNR down to 3.2. Finally, the flat fiber-based scattering system (Fig. 2b, solid line) exhibits a strong narrow peak (FWHM of 0.20 nm) with low background (averaged 0.004), providing a SNR of 32, a tenfold improvement in comparison to the 3D chip and 6.5 times better performance than the MMF-based device.

**Figure 3:**
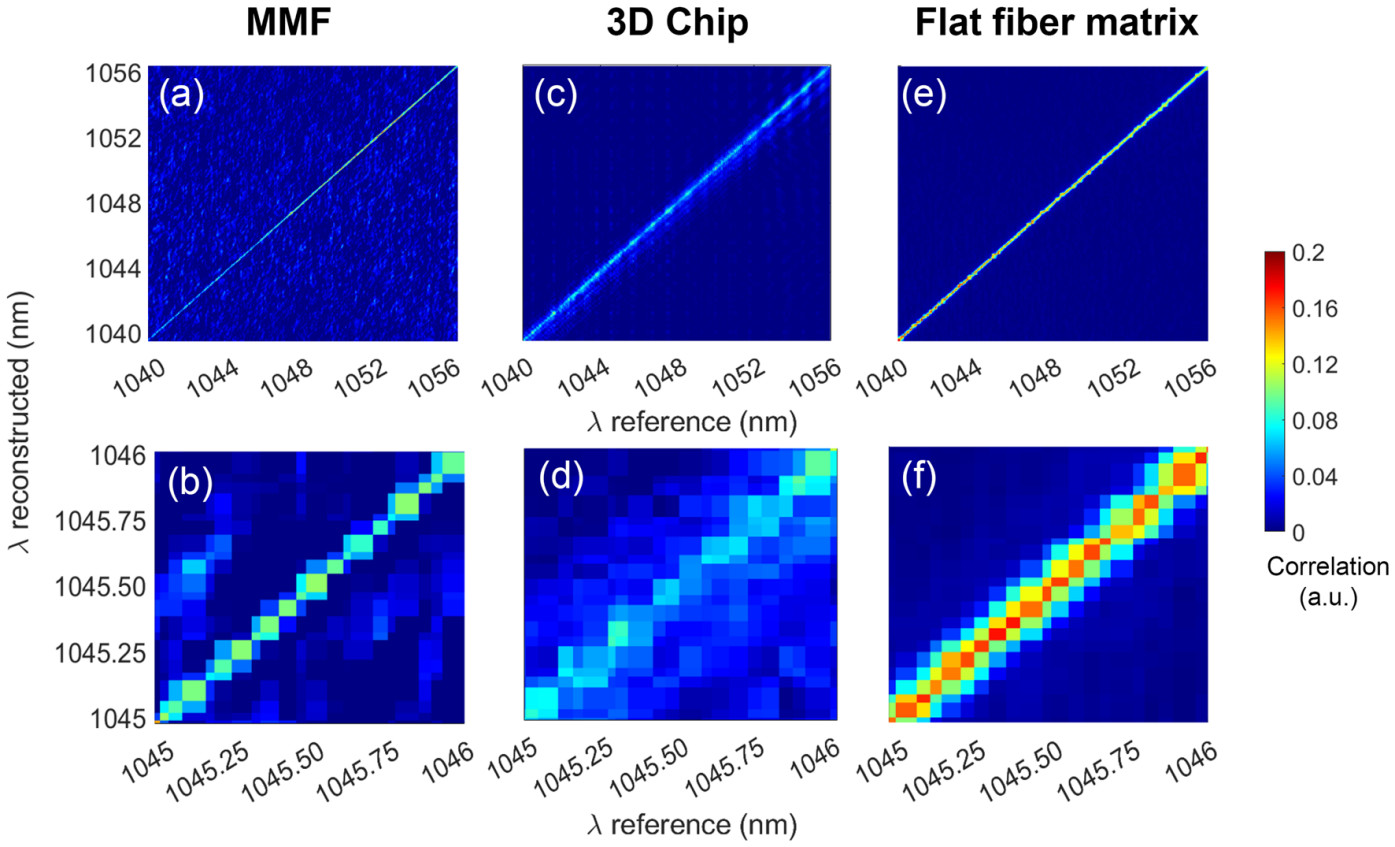
Device operation as wavemeter: correlation matrices between calibration (‘reference’) and testing (‘reconstructed’) wavelengths within the 1040–1056 nm range with spectral separation of 45 pm. Top row sub-figures represent matrices for full spectral range; bottom row graphs show closeups of diagonal over 1 nm wavelength range. Scattering media tested: (**a**, **b**) MMF of 50 cm, (**c**, **d**) 3D scattering chip-based device and (**e**, **f**) flat fiber-inscribed scattering matrix. Colorbar scale refers to correlation value.

To evaluate the device performance and reconstructed spectra shapes over the full wavelength range of 1040–1056 nm, the peak wavelength was swept in approximately 45 pm increments, which is the light source tunability step size, and the obtained results were projected as 2D correlation matrices shown in Fig. 3. Each column corresponds to the reconstructed spectrum for a single wavelength reference input (the aforementioned Fig. 2b graph is essentially a cutline down such a column). In wavemeter operation, the peak gives the reconstructed wavelength value. The full matrix is then computed by linearly incrementing the reference wavelength and repeating the measurement.

Such correlation matrices should ideally have a sharp main diagonal, indicating the peak reconstructed wavelength matches the reference, and low off-diagonal background noise. Although reconstructed wavelength diagonals were present in all three cases, their exact shapes and background levels differed. For the MMF (Fig. 3a,b), the diagonal is the thinnest, which confirms high reconstructive ability with the finest resolution, and a reconstruction error equal to 40 pm. However, its background noise is the highest, with unwanted peaks reaching up to 50% of the diagonal value in places, due to mechanical instability. The 3D scattering chip (Fig. 3c,d) correlation matrix has lower background noise, but its diagonal is broader with a reconstruction standard error of 50 pm and lower peak intensity, making its overall SNR the lowest. This is due to a greater similarity between speckle patterns of adjacent wavelengths. The flat fiber-based scattering array diagonal (Fig. 3e,f) has the greatest peak intensity and also the lowest background noise level, reaching up to 1.5% of the diagonal value. However, since it is slightly broader than the MMF, its standard reconstruction error is marginally worse at 45 pm.

To sum up, the flat fiber-based device combined the stability of the 3D scattering chip with the high resolution of the MMF, resulting in a high-contrast diagonal and low background noise (reconstruction error 45 pm), making it the device with the highest SNR, with strong potential as an excellent reconstructive wavemeter device.

### Long-term stability

Long-term temporal stability is the key characteristic which allows successful commercialization and application of wavemeters. Since the reconstructive device paradigm requires speckles to remain the same during the calibration and reconstruction measurements, this property is even more crucial for scattering speckles-based wavemeters.

To determine such stability, a 60 h long, fixed-wavelength stability experiment was conducted using the same devices as for wavemeter testing, i.e. the MMF, 3D chip and flat fiber scattering matrix. The devices were calibrated and then the laser wavelength was set to a fixed value and left for the duration of the experiment. After the experiment, the differences between the reconstructed and fixed wavelength values were calculated.

**Figure 4:**
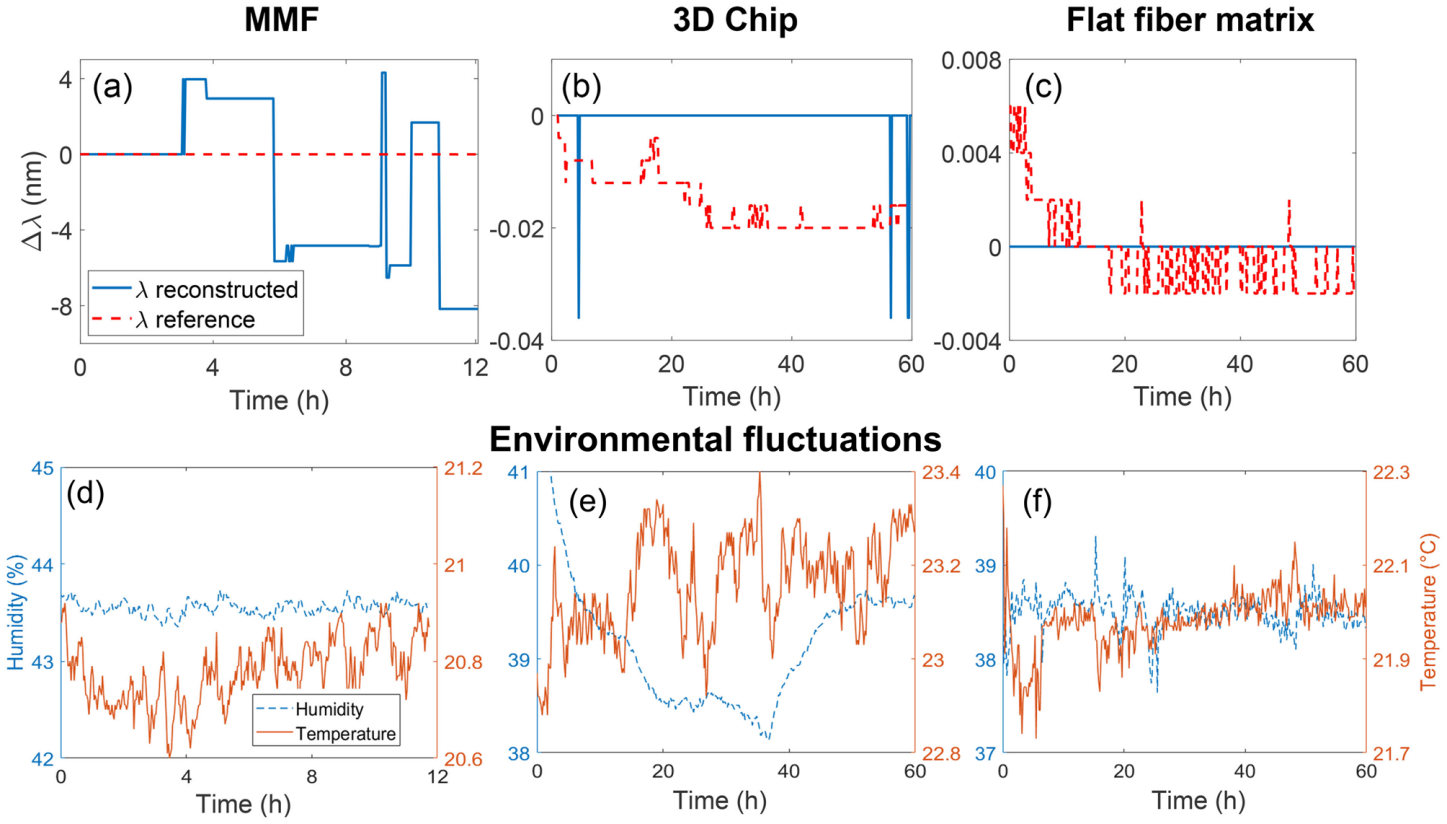
Reconstructed wavelength against time using a fixed input wavelength and fluctuating environmental conditions, comparing temporal stability for wavemeters based on: (**a**, **d**) 50 cm of straight MMF during 12 h (further measurement was not required since instability was already demonstrated by the time of 12 h); (**b**, **e**) 3D scattering chip-based for 60 h and (**c**, **f**) flat fiber-based scattering matrix for 60 h.

Figure 4 shows that the flat fiber-based device is much more stable than those based on MMF and 3D chip. There is no wavelength deviation over the whole period of the experiment ($\Delta \lambda =0$) for the flat fiber-based system, in contrast to the 3D chip (four detunings by 0.038 nm) and MMF (stable up to 3 h, then detunings ranged between $-8$ and 4 nm). The MMF experiments were terminated after 12 h, since high instability was already apparent, instead of the planned 60 h. This confirms the superior stability of the flat fiber-inscribed scattering matrix, which, in tandem with excellent wavemeter operation characteristics, makes it a perfect candidate for compact, cheap, high-resolution and high-stability reconstructive devices. This temporal stability was confirmed even against a fluctuating temperature and humidity. From Fig. 4d it is clear the system was insensitive to environmental fluctuations over a range of at least 37.5–39.5% humidity and 21.7–22.3 °C.

### FBG interrogator

Due to its long-term stability and high resolution, the same flat fiber device can be straightforwardly repurposed as an FBG interrogator. This implementation is based on monitoring speckle patterns arising from microstrain ($\mu \varepsilon$) induced spectral changes whilst stretching/compressing optical fiber with an inscribed FBG.

**Figure 5:**
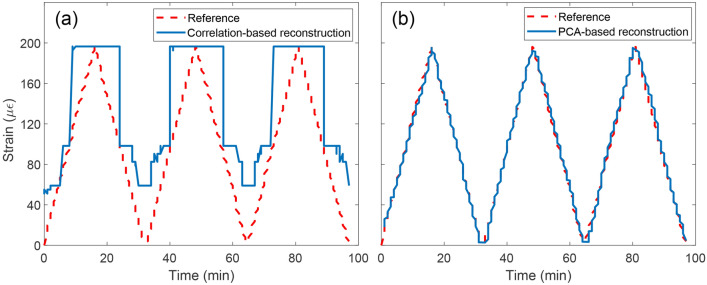
Microstrain detection in the range 0–200 $\mu \varepsilon$ for two reconstruction methods: (**a**) correlation-based reconstruction, showing unreliable recovery of strain and (**b**) PCA-based with reconstruction error of 4 $\mu \varepsilon$. In both cases, the applied strain is ramped linearly up and down (dashed line).

Reconstructing strain from the obtained speckles requires a different approach than the spectral recovery demonstrated in the wavemeter discussion. Since the strain levels are minimal, the same issue can be stated regarding the high similarity between the speckles—the standard reconstructive algorithm solving with the singular value decomposition (SVD)-based correlation equation^10,28^ was not capable of accurate strain reconstruction. The recovered strain resembles binary values between 60 and 200 $\mu \varepsilon$, as shown in Fig. 5a.

Therefore, instead of looking for correlation, the speckles were translated onto a dimension-reduced space which projects only hierarchically ordered changes: from the most dominant to the most negligible, called principal components^10,29–31^. This method, called principal component analysis (PCA) is a fundamental approach to ‘uncover’ masked dependence between the speckles. For the tested microstrain sensing, the first component projection was linear, which is correct since the speckles seemed to be almost identical for the correlation algorithm, while the second principle component unveiled the changes over time which exactly matched the time-varying reference microstrain. Therefore, by collecting speckles and projecting the dataset onto the second principal component space, the strain can be recovered with a maximum standard error of 4 $\mu \epsilon$, which was the limit of the experimental setup due to the translation stage step size (Fig. 5b).

## Discussion

The demonstrated flat fiber scattering matrix-based device breaks the unfavourable compromise between increased stability and reduced spectral resolution, common for all previously reported scattering media-based wavemeters. Here, a wavemeter with a resolution of 45 pm is reported, limited by the wavelength tuning step of the source. By using a finer tuning resolution and a comparable or smaller linewidth for calibration, a higher wavemeter resolution should be achievable. The device is stable for at least 60 h over which there was no single deviation from the reference wavelength. Furthermore, its SNR is approximately 6.5 times better than 50 cm MMF, and 10 times improved compared to the 3D scattering chip device.

On top of that, the system can act as an FBG reflection mode interrogator, tracing microstrain with a step as little as 4 $\mu \epsilon$ in the range 0–200 $\mu \epsilon$ with the standard error equal to the step. The reconstruction was solely based on the speckle pattern changes and no spectral shift information was required. The key requirement is that the detector is sensitive around the Bragg wavelength, but otherwise the system is simple to operate and adaptable for interrogating even complex FBG spectra.

Whilst the peak position of the FBG reflection determines the strain applied, the FBG reflectivity and spectral shape of the reflection may affect the reconstruction capability of the device, thus affecting its resolution. This is due to two factors: (1) the speckle intensity, which is related directly to the reflectivity value and (2) for broadband input light the observed pattern is in fact not a single speckle, but a superposition of all speckles corresponding to the given wavelengths, modified by their intensities. Therefore, the speckle intensity change can be compensated by modifying the sensor exposure parameters (ISO and shutter speed), whilst the outcome of broadband speckle superpositioning may appear ‘blurry’ due to the large number of contributing speckles. As such, the dynamic range is low and its motif pattern is not clearly visible. This may corrupt the reconstruction process and worsen the minimum achievable device resolution.

While the interrogator was calibrated using a single FBG in this demonstration, it can feasibly be adapted to interrogate multiple FBGs in series, provided that their Bragg wavelengths are distinct. This multiplexing feature will be explored as part of future work, where the speckle patterns for each FBG can be calibrated independently, and not necessarily to the same measurand.

## Methods

### Flat fiber fabrication

**Figure 6:**
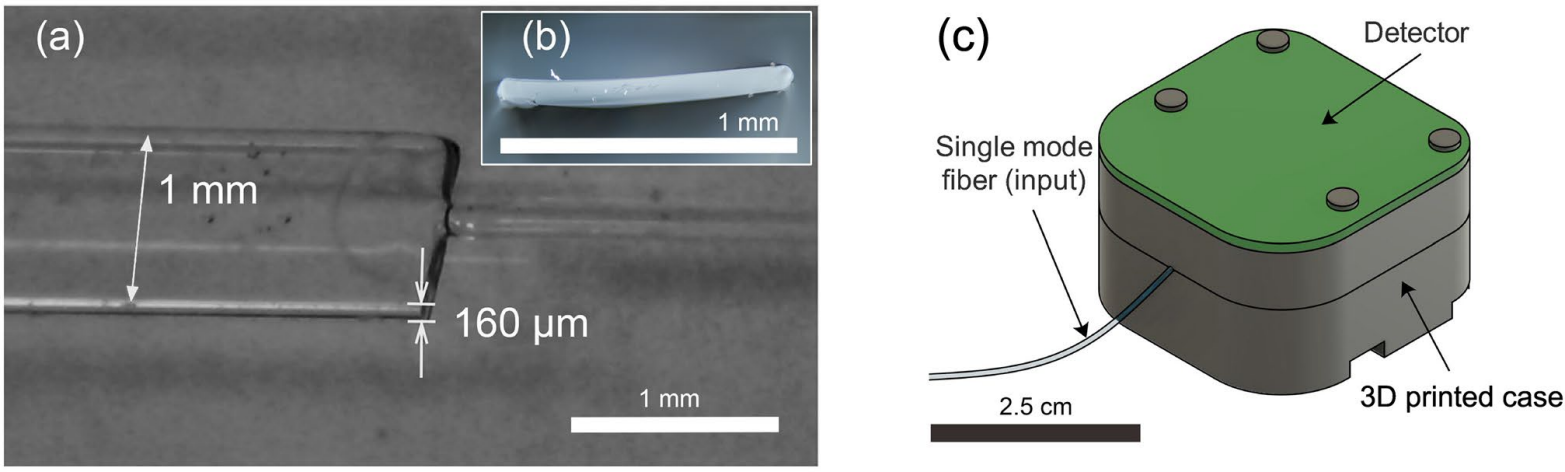
Flat fiber geometry and assembled device: (**a**) splicing point with flat fiber dimensions; (**b**) flat fiber cross-section; (**c**) fully assembled device render. Top circuit board of the detector, 3D printed 2-part case and single mode input fiber are visible. The flat fiber section is enclosed within to eliminate ambient light.

The fused quartz plate preform (2 $\times$ 30 $\times$ 150 mm) was fed into an open throat resistance furnace with a throat diameter of 40 mm. The feed rate was 1 mm/min and the draw rate was 1.4 m/s, controlled by a tractor assembly on the fiber drawing tower. During the draw, the temperature of the furnace was set at 1850 $^\circ$C and these parameters were set to achieve fiber thickness of the order of 100 $\upmu$m and width of 1 mm. It should be noted that the aspect ratio of the preform was 2:30, however this ratio reduced upon draw, to 0.16 ±0.01 due to energy minimization (i.e. surface tension). The 1.5 cm section of the rectangle-shaped (160 $\upmu$m $\times$ 1 mm) extruded flat fiber (Fig. 6a,b) is used as a substrate.

### Scattering matrix inscription

Input single-mode fiber (980 HP, Alker) is spliced to one end of the flat fiber using a laser splicer (LZM-100, Fujikura). The losses at the splicing point are minimal, arising mainly from Fresnel reflection and estimated to be only 0.001%. On this substrate, 15 planar arrays of pseudo-randomized nanovoids, separated by 6.6 $\upmu$m in the vertical direction, are laser-written using a femtosecond laser (Pharos, Light Conversion Ltd., Lithuania) with central wavelength $\lambda =1.03$ $\upmu$m, pulse duration $\tau =200$ fs, repetition rate of $f =200$ kHz and pulse energy lower than 500 nJ. To improve the writing resolution, the second harmonic $\lambda =515$ nm was utilized. Each written plane ($1 \times 0.7$ mm) is constituted of 714 $\times$ 500 voids with a mean spacing of 1.4 $\upmu$m and a randomised change in their position in the range $\pm 0.7$  $\upmu$m alternating in the longitudinal/transverse direction between every plane. Such voids are presented in Fig. 1b. This arrangement was chosen due to the most optimal compromise between fabrication time and scattering efficiency of the device^23^.

### Device assembly

In order to shield the device from ambient light, ensure its integrity (flat fiber is very fragile), align and fix the flat fiber in place and provide a space for mounting the detector, the cover was designed and 3D printed (black Tough PLA, Ultimaker). The laser-inscribed structure was mounted inside. The cover was made of two parts: the upper one housing the detector (Raspberry Pi V2 NoIR camera with lens removed) which is placed 2 mm above the flat fiber, and the bottom one with an inscribed groove for flat fiber mounting. Both halves are then screwed together. The assembled device weighs 100 g with dimensions of $2.5~\times ~2.5~\times ~1.5$ cm (Fig. 6c).

Since the detector (IMX219, Sony) is implemented in Raspberry Pi V2 NoIR camera, the device was fully controlled by Python 3.7 scripts processed by the Raspberry Pi 4B 8GB single-board computer.

### Wavemeter set-up and operation

**Figure 7:**
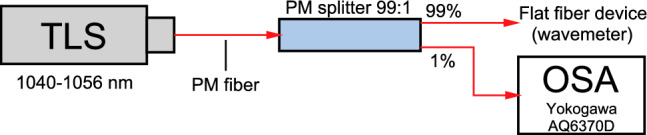
Schematic of wavemeter set-up. Light from tuneable laser source (TLS) is launched into the optical splitter. 99% of the light is transmitted to the flat fiber device, whilst 1% reaches the Yokogawa AQ6370D optical spectral analyzer (OSA) to measure the reference wavelength.

The laser source (tuneable Littman configuration laser diode Thorlabs TLK-L1050M) with lasing range 1040–1056 nm and linewidth of 40 pm was attached via single-mode polarization maintaining (PM) optical fiber to the 99:1 splitter with 99% of power sent to the tested device and the remaining 1% diverted to the reference spectrum analyzer (Yokogawa OSA AQ6370D) (Fig. 7). Due to the nature of the laser source wavelength tuning motor, it was impossible to obtain a fixed wavelength tuning step (it ranged from 35 to 55 pm). Therefore, its average value of 45 pm was defined as the tuning step value.

The experiment was run in two phases: the calibration and the data collection. For the first phase, the peak of the wavelength was read from the Yokogawa OSA simultaneously with the speckle capturing (ISO=50, shutter speed=0.4 s). This operation was repeated for 340 steps, changing the wavelength from 1040 to 1056 nm in increments of $\sim$ 45 pm.

Once the calibration data collection was completed, the source was re-tuned to 1040 nm and the operation was repeated. As a result, the data consists of two sets of 340 speckles (‘calibration’ and ‘testing’) accompanied by readings of wavelength peaks from the OSA during calibration. This process was performed for three different scattering media, to demonstrate the unique features of flat fiber-based system: 50 cm of straight rail-guided MMF (Thorlabs, FG105LCA), the 3D scattering chip and the flat fiber-based scattering matrix.

### FBG interrogation set-up and operation

The FBG interrogation was done in reflection mode—the Bragg resonance wavelength is reflected back and detected as a speckle by the demonstrated device. The shift of the resonance wavelength is related to the strain by the following equations:

1 $$\begin{aligned} \varepsilon =\frac{L-L_{0}}{L_{0}}=\frac{\Delta L}{L_{0}} \end{aligned}$$

2 $$\begin{aligned} \frac{\lambda _{B}-\lambda _{B0}}{\lambda _{B0}}=\frac{\Delta \lambda }{\lambda _{B0}}=0.79\varepsilon \end{aligned}$$

where $\epsilon$ is the strain, $L_{0}$ the initial length of fiber before applying strain, *L* the fiber length after applying strain, $\lambda _{B0}$ the idle Bragg resonance wavelength (no strain), $\lambda _{B}$ the shifted Bragg resonance wavelength after applying strain.

**Figure 8:**
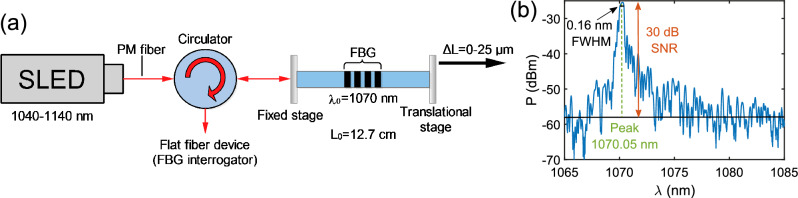
(**a**) Schematic of FBG interrogation set-up. The light from superluminescent diode (SLED) is launched into the optical circulator then transmitted to the FBG with the Bragg resonance wavelength of 1070 nm. When strain is applied (in the range 0–25 $\upmu$m), the resonance wavelength shifts and the reflected wavelength comes back through the circulator to the flat fiber device which generates speckles from the reflected wavelength and therefore acts as the interrogator. (**b**) Reflected power spectrum of the tested FBG, showing the peak signal is 30 dB higher than the background noise level.

The superluminescent diode (SLED) SLD-1080-30-PM-100 light source from Innolume, with a spectral range of 1040–1140 nm, was connected to the optical circulator via PM fiber. Then, the light was sent to the in-house made 3 cm long FBG inscribed in PS980 optical fiber with a reflection peak at 1070 nm, 0.16 nm FWHM linewidth and 30 dB reflection signal-to-noise ratio (Fig. 8). The FBG was mounted on linear translation stages (MAX381, Thorlabs). One of the stages remained at a fixed position during the measurement, whilst the other was displaced in the fiber’s longitudinal direction in the range 0–25 $\upmu$m with a minimum repeatable step of 0.5 $\upmu$m (DRV001 step-motor limit). The initial length of the fiber containing the inscribed FBG equaled 12.7 cm. The reflected FBG spectrum was launched back through the circulator and projected to the tested flat fiber-based device, which in turn converted the spectrum into speckles (Fig. 8). Similarly to the wavemeter operation, the experiment consisted of calibration and measurement phases.

During the calibration, 50 speckles were collected, corresponding to stretching the fiber from 0 to 25 $\upmu$m in 0.5 $\upmu$m increments, as well as the feedback from the device encoder of how much the stage was displaced for every speckle (an analogy to the OSA reference for wavemeter operation). The stage translations were converted to the strain values as per Eq. (1).

For the measurement, 303 speckles were collected by ramping the stage 3 times from 0 to 25 $\upmu$m and back to 0 $\upmu$m with the minimum possible step of 0.5 $\upmu$m (Fig. 5).

### Reconstructive algorithms

Capturing speckle patterns is just the first step of device operation. Due to the nature of the operating paradigm, the device performance is strongly related to both its physical design (how the speckles are generated and captured) and processing algorithms (processing data to recover the output). Therefore, it is important to understand the two methods involved in the reconstruction process.

#### Solving correlation equation by singular value decomposition (SVD)

During the calibration process, the device captures speckles of the different known wavelengths as 3D RGB 8-bit matrices, stored as .png files. Upon completion, each image is grayscaled to a 2D matrix with luminance information and then vectorized (converted into single-column by stacking each consecutive column from the 2D matrix to the bottom of the previous one) and normalized (divided by 255 so each matrix would contain numbers in the range 0–1). By doing so, the calibration *C* matrix can be constructed, in which each column corresponds to the single speckle vector at each known wavelength.

The same operation can be repeated for the measured (‘unknown’) data—the speckle vector can be obtained by grayscaling, vectorizing and normalizing the initial speckle pattern. Having both the calibration matrix *C* and measured pattern vector *P* (of the unknown spectrum under test), it is obvious there is one more vector which carries the information of how much of each calibration (i.e. of each ‘reference’) vector is embedded in the unknown pattern vector (Eq. 3).

3 $$\begin{aligned} P=C\cdot {}S \end{aligned}$$

This vector is called the spectrum *S* and its values represent the weights of each reference speckle (and therefore the wavelengths) which constituent the unknown measured speckle. The spectrum can be projected by plotting its values versus calibration wavelengths. To reconstruct it, the equation needs to be rearranged (Eq. 4):

4 $$\begin{aligned} S=C^{-1}\cdot {}P \end{aligned}$$

where $C^{-1}$ is the inversion of the calibration matrix (*C*). However, the challenge arises since for the vast majority of the cases (speckle area${\gg}$number of calibration wavelengths (speckles)) so the calibration matrix is non-square and by definition, it is impossible to obtain its inverse, since it does not exist. Fortunately, there is a solution: the Moore–Penrose pseudo-inverse, which can be conveniently computed using singular value decomposition (Eq. 5)^10,12^.

5 $$\begin{aligned} C=U\Sigma {}V^{T}\leftrightarrow C^{-1}=V\Sigma ^\prime {}U^{T} \end{aligned}$$

where $U,\Sigma {},V$ are the SVD matrices from decomposing matrix *C*, $^{T}$ the transposition operation (flipping rows and columns of a matrix), $C^{-1}$ the pseudo-inverse of *C*, and $\Sigma ^\prime$ the pseudo-inverse of $\Sigma {}$, obtained by reciprocating its diagonal and then transposing the obtained matrix.

SVD not only solves the non-square matrix inversion problem, but allows denoising (truncation) of the recovered spectra, effectively removing the noise which had a very high impact on the reconstruction, since the smallest values of the diagonal of $\Sigma {}$ matrix would be reciprocated, therefore having the highest value within the obtained pseudo-inverse. In order to find the optimal truncation threshold (effective denoising), the solution proposed by Gavish and Donoho^32^ was applied for the experimental data.

#### Principal component analysis (PCA)

The principal component analysis (PCA) can be applied if the speckle changes are too small to be detected via the correlation equation (which would see small or no change at all, even if in fact there is a significant change in the sensed quantity like strain applied to the FBG where the flat fiber device was used as the interrogator (Fig. 5). From its definition, the PCA can be interpreted as the statistical representation of the SVD which is a bedrock for data-dimensionality reduction, uncovering the low-dimensional data pattern or trend which cannot be otherwise recovered^33,34^. Essentially, it can be used to project the data (speckle vectors) into dimension-reduced space and observe the hierarchically-ordered (describing the change from the most to the least dominant one) set of values which describe this projection, called principal components.

The practical realization of the PCA idea is based on the SVD, but with additional data pre-processing operations, since now this method is a statistical representation of the SVD. These additional steps are: (1) transposing data matrix (number of rows corresponds to the number of speckle observations and number of columns to the number of pixels in each speckle); (2) computation of an average observation row vector (Eq. 6); (3) creation of an average matrix by multiplying column vector of ones with an average observation row vector (Eq. 7); (4) the average matrix is subtracted from the data matrix, therefore generating mean-centered data matrix (Eq. 8).

6 $$\begin{aligned} \bar{x}=\frac{1}{n}\sum _{j=1}^{n} x_{j} \end{aligned}$$

7 $$\begin{aligned} \bar{X}=\begin{bmatrix} 1_{1}\\ 1_{2}\\ \vdots \\ 1_{n} \end{bmatrix}\bar{x} \end{aligned}$$

8 $$\begin{aligned} B=X-\bar{X} \end{aligned}$$

where *X* is the data matrix with *n*-rows, with each row corresponding to single speckle observation (speckle vector), $\bar{x}$ the average row of data matrix, $x_{j}$ the *j*th row of data matrix, $\bar{X}$ the average data matrix, and *B* the mean-centered data matrix. Now, the SVD can be applied to the data-centered matrix *B* (Eq. 9)

9 $$\begin{aligned} B=U\Sigma {}V^{T} \end{aligned}$$

which in turn leads to the principal components matrix and loadings vector:

10 $$\begin{aligned} P_{PCA}=U\Sigma {} \text { and } L_{loads}=V \end{aligned}$$

where $P_{PCA}$ is the matrix containing *n* principal components sets for all variables (total pixel speckle number), and $L_{loads}$ the matrix showing for each column the linear combination of the original variables from which the principal components are constructed, denoting how much of total data variance is being captured by given principal component set.

The data for final interpretation can be obtained by performing the projection operation (Eq. 11).

11 $$\begin{aligned} X_{Projected}=P_{PCA}^T\cdot {}X \end{aligned}$$

where $X_{Projected}$ is the projected data matrix to the given principal components set, *X* the initial unprocessed data matrix, and $P_{PCA}(n)$ the selected vector (set of the principal components) from the principal components matrix $P_{PCA}$. By observing the resulting data projection, the masked relationships can be uncovered, which allowed near-perfect microstrain reconstruction, in contrast to the approach of solving the correlation equation on its own (Fig. 5).

## Data Availability

The datasets generated and analysed during the current study are available in the University of Southampton repository, https://doi.org/10.5258/SOTON/D2441.
